# Non-pharmaceutical interventions for COVID-19: a systematic review on environmental control measures

**DOI:** 10.1098/rsta.2023.0130

**Published:** 2023-10-09

**Authors:** Anagha Madhusudanan, Christopher Iddon, Muge Cevik, James H. Naismith, Shaun Fitzgerald

**Affiliations:** ^1^ Isaac Newton Institute for Mathematical Sciences, CB3 0EH, Cambridge, UK; ^2^ Department of Civil, Environmental and Geomatic Engineering, University College London, WC1E 6BT, London, UK; ^3^ Department of Infection and Global Health, School of Medicine, University of St Andrews, KY16 9TF, St Andrews, UK; ^4^ Roslind Franklin Institute, Harwell Campus, Didcot OX11 0QX, UK; ^5^ Department of Engineering, University of Cambridge, CB2 1PZ, Cambridge, UK

**Keywords:** ventilation, COVID-19, disinfection, air cleaning, occupancy

## Abstract

The purpose of this review was to identify the effectiveness of environmental control (EC) non-pharmaceutical interventions (NPIs) in reducing transmission of SARS-CoV-2 through conducting a systematic review. EC NPIs considered in this review are room ventilation, air filtration/cleaning, room occupancy, surface disinfection, barrier devices, ${\mathrm{CO}}_{2}$ monitoring and one-way-systems. Systematic searches of databases from Web of Science, Medline, EMBASE, preprint servers MedRxiv and BioRxiv were conducted in order to identify studies reported between 1 January 2020 and 1 December 2022. All articles reporting on the effectiveness of ventilation, air filtration/cleaning, room occupancy, surface disinfection, barrier devices, ${\mathrm{CO}}_{2}$ monitoring and one-way systems in reducing transmission of SARS-CoV-2 were retrieved and screened. In total, 13 971 articles were identified for screening. The initial title and abstract screening identified 1328 articles for full text review. Overall, 19 references provided evidence for the effectiveness of NPIs: 12 reported on ventilation, 4 on air cleaning devices, 5 on surface disinfection, 6 on room occupancy and 1 on screens/barriers. No studies were found that considered the effectiveness of ${\mathrm{CO}}_{2}$ monitoring or the implementation of one-way systems. Many of these studies were assessed to have critical risk of bias in at least one domain, largely due to confounding factors that could have affected the measured outcomes. As a result, there is low confidence in the findings. Evidence suggests that EC NPIs of ventilation, air cleaning devices and reduction in room-occupancy may have a role in reducing transmission in certain settings. However, the evidence was usually of low or very low quality and certainty, and hence the level of confidence ascribed to this conclusion is low. Based on the evidence found, it was not possible to draw any specific conclusions regarding the effectiveness of surface disinfection and the use of barrier devices. From these results, we further conclude that community agreed standards for well-designed epidemiological studies with low risk of bias are needed. Implementation of such standards would enable more confident assessment in the future of the effectiveness of EC NPIs in reducing transmission of SARS-CoV-2 and other pathogens in real-world settings.

This article is part of the theme issue ‘The effectiveness of non-pharmaceutical interventions on the COVID-19 pandemic: the evidence’.

## 1. Background

Understanding of SARS-CoV-2 transmission has evolved significantly since the beginning of the pandemic. The rapid spread of SARS-CoV-2 worldwide presented a unique challenge [1,2]. For instance, in the UK the official number of cases in March 2020 was doubling roughly every 4 days [3]. As public health officials and governments recognized the threat that COVID-19 posed, slowing the spread of infection became a priority to save lives [4] (table 1).

**Table 1.** Nomenclature.

ACH	air changes per hour
AOR	adjusted odds ratio
CI	confidence interval
COVID-19	coronavirus disease 2019
EC	environmental control
EMG	environmental and modelling group
GRADE	grading of recommendations, assessment, development and evaluations
HCW	healthcare workers
HEPA	high-efficiency particulate arrestence
NPI	non-pharmaceutical intervention
OR	odds ratio
ppm	parts per million
qRT-PCR	quantitative reverse transcription polymerase chain reaction
RNA	ribonucleic acid
ROBINS-I	risk of bias in non-randomized studies—of interventions
SAGE	scientific advisory group for emergencies—UK
SARS-CoV-2	severe acute respiratory syndrome-related coronavirus 2
UVGI	ultraviolet germicidal irradiation

Many countries implemented isolation of imported cases of COVID-19 and their contacts, but by late February 2020 cases of community transmission with no links to travel were identified in the UK and many other countries [5]. The rapidity of the spread and the consequences of exponential growth in case numbers meant the potential consequences were escalating rapidly. Like many countries, the UK Government took the unprecedented step of implementing a so-called lockdown on 23 March 2020, legally a stay at home order to slow the spread of infection and reduce the impact on health services [4]. However, transmission is complex and depends on many factors including environmental ones [6–8]. Environmental interventions were therefore also put in place to try and control the spread of SARS-CoV-2.

Although highly effective in reducing transmission, the adverse social and economic consequences associated with lockdown [9] meant that this measure could not be sustained for prolonged periods. Consequently, there was a desire to implement other control measures that could contribute to preventing a resurgence in the number of infected people. Control measures implemented during the pandemic were aimed at combating either touch (fomite), direct person-to-person, short- and long-range aerosol, or a combination of these modes of transmission.

This review of the impact of environmental controls (ECs) covers ventilation, occupancy, disinfection and air filtration. ECs are defined as measures which were intended to alter the potential contamination level of surfaces, imposed barriers to person-to-person contact, and modified the air within buildings. By focusing on transmission rather than surrogate markers such as virus detection in the environment, the review seeks to identify the effectiveness of measures in terms of reduction of transmission in real-life situations. While modelling and experimental studies help inform our understanding about the role of various ECs, direct extrapolation of their findings for humans in real-life situations is limited.

## 2. Methodology

### (a) Search strategy

Systematic searches of databases from Web of Science, Medline, EMBASE, preprint servers MedRxiv and BioRxiv were conducted in order to identify studies reported between 1 January 2020 and 1 December 2022. All articles reporting on the effectiveness of ventilation, air filtration/cleaning, room occupancy, surface disinfection, barrier devices, ${\mathrm{CO}}_{2}$ monitoring and one-way systems in reducing transmission of SARS-CoV-2 were retrieved and screened. The search was conducted using medical subject headings (MeSH) terms (electronic supplementary material).

### (b) Study selection

Studies were included in the review if they (i) reported on the transmission of SARS-CoV-2 in humans or animals and (ii) reported how transmission is impacted by the implementation of the following EC NPIs: ventilation, air cleaning devices, surface disinfection, room occupancy modification, barrier devices, ${\mathrm{CO}}_{2}$ monitoring and one-way systems. Papers were excluded if they

1. did not consider the transmission of SARS-CoV-2 between humans or animals
2. did not include a comparison between groups that implemented the NPI and groups that did not
3. were modelling studies with no original data
4. were experimental studies that used model aerosols with no SARS-CoV-2 virus
5. were studies on environmental sampling alone
6. did not include original research such as review papers etc.
7. were not published in English.

### (c) Data extraction

Three authors (A.M., C.I. and M.C.) screened the retrieved articles based on the title and abstract of the references. The obtained references were screened a second time by one reviewer (A.M.) to further exclude references based on criteria 3–6 listed above in §2b. A second reviewer (C.I.) reviewed 5% of the exclusions. Four reviewers (A.M., C.I., M.C. and S.F.) then performed a full text review in order to select the final papers based on the inclusion/exclusion criteria in §2b. Each paper selected for full text review was reviewed first by one reviewer (A.M.) and inclusion/exclusion decisions that were not straightforward were reviewed by a second reviewer (C.I., M.C. or S.F.). Disagreements were then subsequently resolved by a third reviewer. The following variables were noted in the final papers: country, setting, environmental NPI implemented, sample size, SARS-CoV-2 transmission results and other factors associated with transmission. Where available the following data were also summarized: measurements related to the environmental NPI considered (air changes per hour (ACH) or ${l\;s}^{-1}$ for ventilation, area/volume available per person for occupancy); the number of infected subjects in the cases with and without the NPI implemented.

### (d) Risk of bias in included studies

Three authors (M.C., A.M. and C.I.) made an assessment of methodological study quality using a Cochrane ‘risk of bias’ tool for non-randomized studies. Risk of bias assessment for each study using ROBINS-I is included in table 2. GRADE was then used to assess evidence quality for each review, table 3. Evidence quality was downgraded from ‘high quality’ by one level for each serious issue identified in the domains of risk of bias (imprecision, indirectness, inconsistency and publication bias), and upgraded by one level for each factor which increased the quality of evidence including the domains of large magnitude of effect, residual confounding and dose–response gradient.

**Table 2.** ROBINS-I assessment of the included references—domain 1 pre-intervention confounding; domain 2 selection bias; domain 3 classification of intervention; domain 4 post-intervention confounding; domain 5 missing data; domain 6 bias in measurement; domain 7 reporting bias. Overall risk of bias assessment 0, No information; 1, low; 2, moderate; 3, serious;4, critical; EC NPI; V, ventilation, O, occupancy; ACD, air cleaning device; D, disinfection; S, screens.

study	domain 1	domain 2	domain 3	domain 4	domain 5	domain 6	domain 7	EC NPI
Li *et al.* [10]	3	3	3	3	2	2	2	V
Oginawati *et al.* [11]	4	1	1	2	2	3	2	V,O
Walshe *et al.* [12]	3	2	3	3	2	1	2	V,O
Ou *et al.* [13]	3	2	2	3	2	2	2	V
Pakora *et al.* [14]	4	3	3	4	3	4	2	V
Nabirova *et al.* [15]	4	3	3	3	3	4	2	V
Baumgarte *et al.* [16]	4	3	2	4	2	3	2	V,O
Gettings *et al.* [17]	4	3	3	4	3	4	2	V,ACD,S
Feathers *et al.* [18]	3	2	4	3	2	4	2	V
Dancer *et al.* [19]	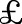	2	4	3	2	4	2	V
Guedes *et al.* [20]	4	3	3	4	3	4	2	V,D
Wang *et al.* [21]	4	3	3	4	3	4	2	V,D
Atnae *et al.* [22]	4	3	2	4	3	4	2	D
Kerai *et al.* [23]	4	3	3	4	3	4	2	D
Telford *et al.* [24]	4	3	3	4	3	3	2	O,D
Szablewski *et al.* [25]	4	3	3	4	2	2	2	O
Cheng *et al.* [26]	4	4	4	4	2	3	2	ACD,O
Fischer *et al.* [27]	1	1	1	2	2	1	2	ACD
Zhang *et al.* [28]	1	1	1	2	2	1	2	ACD

**Table 3.** GRADE assessment of the included references—factors that can reduce quality: domain 1 limitations in study design or execution; domain 2 inconsistency of results; domain 3 indirectness of evidence; domain 4 imprecision; domain 5 publication bias; factors that can increase the quality: domain 6 large magnitude of effect; domain 7 residual confounding; domain 8 dose–response gradient. Assessment ↓–assessed down 1 or 2 levels; ↑–assessed up 1 or 2 levels; - –no effect.

	factors that can reduce quality				factors increase the quality			
study	domain 1	domain 2	domain 3	domain 4	domain 5	domain 6	domain 7	domain 8
ventilation	↓2	↓2	↓1	↓2	—	—	—	—
air cleaning								
devices	↓2	↓2	↓1	↓2	—	—	—	—
occupancy	↓2	↓2	↓1	↓2	—	—	—	—
disinfection	↓2	↓1	↓2	↓2	—	—	—	—

## 3. Results

**Figure 1.**
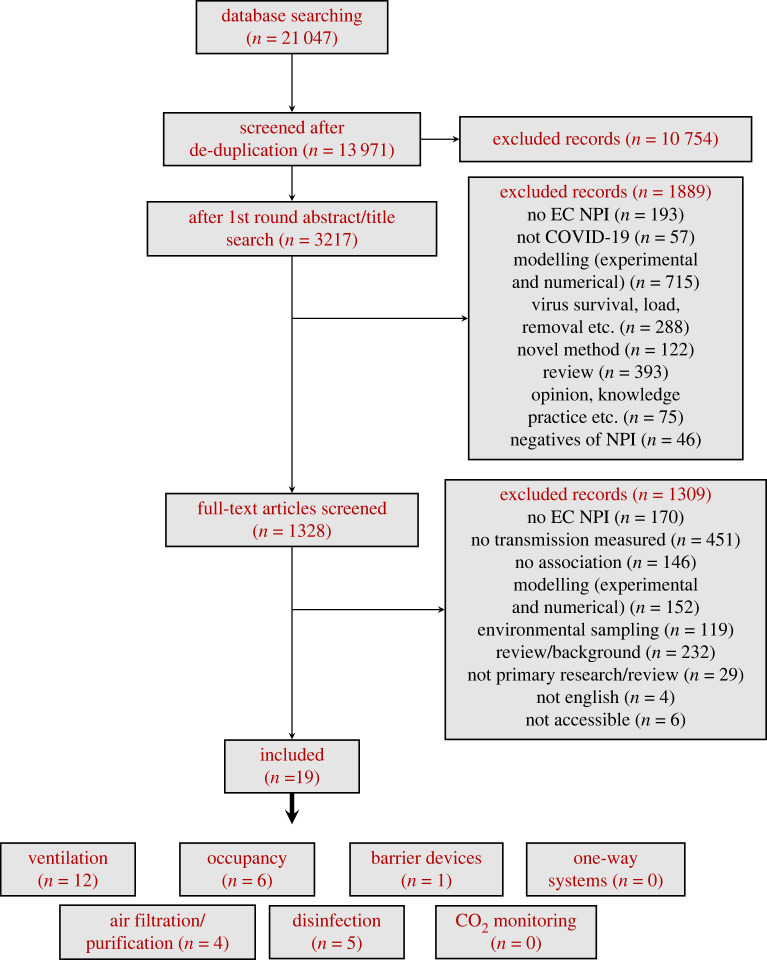
Flowchart representing the procedure followed for selecting the studies included in this review.

In total, 13 971 unique articles were identified in the systematic search. A total of 3217 of these were retrieved based on initial title and abstract screening. Further screening of the titles and abstracts based on the inclusion/exclusion criteria in §2b reduced the number of articles for full-text review to 1328. Only 19 of these studies met the inclusion criteria for the review; those which had been initially identified through pre-print servers were subsequently published following peer review, and it was the peer reviewed version which was included. For a detailed description of the number of references at each stage, see the Prisma flowchart in figure 1. Overall, the 19 studies included here provide evidence for the effectiveness (or ineffectiveness) of the following NPIs: ventilation (n=12) [10–21]; air filtration/air cleaning devices (n=4) [17,26–28]; surface disinfection (n=5) [20–24]; room occupancy (n=6) [11,12,16,24–26]; and screens/barriers (n=1) [17]. No evidence was found on the use of ${\mathrm{CO}}_{2}$ monitoring or one-way systems in reducing transmission. These 19 studies covered a range of settings: healthcare facilities (n=6) [18, 20,22–24]; residential (*n*=3) [10,11,21]; meat-processing plants (n=2) [12,14]; school classrooms (n=2) [16,17]; an overnight camp (n=1) [25]; a bus (n=1) [13]; a restaurant (n=1) [26]; and an oilfield (n=1) [15]. Two studies were laboratory studies using animal models (n=2) [27,28]. Table 4 in appendix provides a summary of the information extracted from these 19 references.

All of the included studies were found to have critical or serious risk of bias in at least one domain, see §4.

### (a) Ventilation

Among the 12 studies that considered the effectiveness of ventilation on the transmission of SARS-CoV-2: (i) six studies provided evidence suggesting improved ventilation decreases SARS-CoV-2 transmission, (ii) three studies found no association between improved ventilation and transmission and (iii) three studies considered the impact of a combination of NPIs that included ventilation, therefore making it difficult to determine the effect on transmission of ventilation alone.

*Positive association*: Six (of 12) studies provided evidence suggesting improved ventilation decreases SARS-CoV-2 transmission. Only two of these studies directly measured ventilation rates, either experimentally (Walshe *et al.* [12]) or using a combination of experiments and computational fluid dynamics (CFD) (Ou *et al.* [13]). These studies present the strongest evidence on the effectiveness of increased ventilation [12,13]. Walshe *et al.* [12] measured the ventilation rates in two areas of a meat-processing plant that experienced different rates of transmission during a SARS-CoV-2 outbreak. These areas were: (i) the ‘boning hall’ which experienced higher transmission and had a measured ACH ≈0.4--0.5; and (ii) the ‘abattoir’ which experienced lower levels of transmission and had a measured ACH ≈8. Ou *et al.* [13] measured the ventilation rates in two buses with slightly different secondary infection rates after an infected index case travelled in them: (i) 7/46 (15%) passengers tested positive after travelling in bus B1 which had a ventilation rate of $1.72\;{l\;s}^{-1}\;\text{per person}$ and (ii) 2/17 (12%) passengers were infected after travelling in bus B2 which had a ventilation rate of $3.22\;{l\;s}^{-1}\;\text{per person}$. In both of these studies, the measurements showed that the setting with increased ventilation had lower transmission. However, there were confounding factors that reduce confidence in this conclusion. Most importantly, Walshe *et al.* [12] noted that in addition to the different ventilation rates in the boning hall and abattoir, the occupancy rates were also different, with higher transmission also being associated with the higher occupancy area (see §3b). Therefore, the study in fact showed that the combination of improved ventilation and lower occupancy was associated with lower transmission. This limits the quantification of the effect of ventilation alone.

Additionally, the viral load present in the boning hall (where the outbreak originated) may have been different from that in the abattoir, which could have impacted the differences in secondary infection rates. In the study of the two buses (Ou *et al.* [13]), the exposure times to the index case were reported to be different, an important factor that could also have affected the secondary infection rates.

In two of the other studies which showed decreased transmission with increased ventilation (Oginawati *et al.* [11] and Baumgarte *et al.* [16]), ventilation rate was not directly measured; instead, the ventilation rate was simply inferred from the dimensions of the setting (such as the area of the available windows, doors etc.) and expected flow rate. Oginawati *et al.* [11] considered secondary infection rates in 38 houses and showed a statistically significant correlation between inferred improved ventilation and lower secondary infection rates. Baumgarte *et al.* [16] reported on an outbreak where the index case, a teacher, taught in four different classrooms. The classroom that had the highest secondary infection rates was found to have an inferred lower rate of ventilation than the other three classrooms. However, the classes were taught on two different days, the personal protective behaviour of the teacher was different on these two days (with the least impacted classrooms having been taught on the day when the teacher had improved personal protective behaviour), and the viral load present in the respiratory tract of the teacher might also have been different on the two days.

The presence/absence of a ventilation system was assessed through questionnaires in the studies by Gettings *et al.* [17] and Pokora *et al.* [14]. Gettings *et al.* [17] studied 169 schools and found that SARS-CoV-2 transmission was 35% lower in classrooms which implemented air dilution methods of ventilation (opening doors, opening windows or using fans) alone. They also further showed that in classrooms which incorporated air dilution methods along with air cleaning devices (high-efficiency particulate arrestance (HEPA) filter and/or ultra–violet germicidal irradiation (UVGI)) transmission was reduced by as much as 48% (see also §i). Pokora *et al.* [14] analysed 22 meat and poultry plants and concluded that the presence of a ventilation system statistically reduced the chances of workers testing positive for SARS-CoV-2.

*No association*: Three (of 12) studies showed no significant association between the presence of a ventilation system and SARS-CoV-2 transmission. In all three studies, data were collected through questionnaires or interviews. Wang *et al.* [21] studied secondary infection rates among 124 families. Guedes *et al.* [20] considered healthcare settings of Hemodialysis units and studied the association between different NPIs and the presence of COVID-19 clusters. Nabirova *et al.* [15] considered oilfield workers who lived in camps, and compared different variables associated with workers from the same camp who tested positive and negative for SARS-CoV-2. In the study by Wang *et al.* [21], there was a SARS-CoV-2 patient present in each family but there was no record of the amount of virus being emitted by the index case. Similarly, in the study by Guedes *et al.* [20], there was no record of the infectiousness of the index cases nor whether clusters were all derived from a single infector (there was no sequencing) or just a number of non-associated infections from the community; there were also multiple NPIs applied in these settings. In all three studies, ventilation was one of several different factors considered and although no significant association was found between the presence of a ventilation system and COVID-19 outcomes confidence in all these studies is weak, which is borne out in the GRADE ROBINS-I analysis, tables 2 and 3.

*Unclear association*: Three (of 12) studies considered ventilation in combination with a range of other NPIs (Li *et al.* [10] Dancer *et al.* [19] and Feathers *et al.* [18]) and do not enable one to determine the effect of ventilation alone, although they provide an understanding of how different NPIs can work together to affect transmission. Li *et al.* [10] found that in a residential setting increased ventilation (measured using ${\mathrm{CO}}_{2}$ tracer decay) in combination with lower adherence to masking (adherence qualitatively assessed) was associated with increased transmission.

Feathers *et al.* [18] and Dancer *et al.* [19] studied outbreaks in healthcare settings, where after detecting the outbreak, multiple NPIs which included ventilation were implemented. Case numbers decreased after implementation of the NPIs. However, this decrease occurred against declining background infection rates and the vaccination status of the population studied in Dancer *et al.* [19] changed over the study period. These reasons, along with the fact that multiple NPIs (including screening, masking etc.) were simultaneously applied, preclude confident conclusions regarding ventilation alone from these studies.

#### (i) Air cleaning devices

All of the four studies that considered the effectiveness of air cleaning devices reported evidence for reduced viral transmission [17,26–28]. However, in one of the reports by Cheng *et al.* [26], the differences between the populations studied made it hard to establish a clear association.

*Positive association*: The only controlled laboratory experiments identified in this review were undertaken by Fischer *et al.* [27] and Zhang *et al.* [28], and they showed a positive association between air cleaning and reduced SARS-CoV-2 transmission. Both studies involved animal models. Fischer *et al.* [27] separated two infected donor hamsters from two naive hamsters. The boxes housing each group were connected by a tube that either (i) had a UV-C light treatment fitted inside it for one experiment or (ii) no UV-C light fitted inside for the other one. SARS-CoV-2 genomic material was found in the naive hamsters occupying the box without UV-C light treatment fitted to the connecting tube, demonstrating that they had become infected. By contrast, SARS-CoV-2 genomic material was not found in the group with UV-C light treatment. Zhang *et al.* [28] showed that, in the presence of three infected hamsters, a negative ionizer protected three naive hamsters in separate cages from aerosol transmission. They also showed that the ionizer provided no protection for direct-contact transmission [28]. However, it is important to note that the concentration of SARS-CoV-2 in the air was not measured in either of these studies, and the dose–response of animal models is likely to be different to that of humans.

Gettings *et al.* [17] (already mentioned in §3a) studied 169 schools using questionnaires and showed that while the presence of air dilution methods of ventilation reduced SARS-CoV-2 transmission by 35%, when air dilution was combined with air cleaning devices (HEPA filter and/or UVGI) the transmission was reduced by as much as 48% [17]. However, the ventilation rates were not measured in this study and it was also conducted over a short time period.

*Unclear association*: Cheng *et al.* [26] studied two outbreaks in restaurants R1 and R2 and found that lower transmission occurred in the restaurant with air cleaning devices (2.6% secondary infection rate in R2 from one index case) than in the restaurant with no air cleaning devices (33.7% secondary infection rate in R1 from an unknown number of index cases). However, the diners in restaurant R2 were vaccinated whereas the diners in R1 were not [26], the number of index cases present in R1 was not reported in the study, and the reported occupancy rates in R1 and R2 were different. Consequently, it is not possible to establish the effect of air cleaning devices alone from this study.

### (b) Occupancy

Among the six studies that considered the impact of room occupancy on SARS-CoV-2 transmission, (i) four studies provided evidence suggesting that decreasing room occupancy leads to a reduction in SARS-CoV-2 transmission, (ii) one study concluded that there is no association between room occupancy and transmission and (iii) one study showed unclear association.

*Positive association*: Walshe *et al.* [12] (see also §3a) considered two regions of a meat factory and found that the area with higher occupancy ($5.1\;m^{2}$ floor area per person in the ‘boning hall’) experienced higher transmission than the area with lower occupancy ($15.2\;m^{2}$ floor area per person in the ‘abattoir’). However, the abattoir had a higher ventilation rate than the boning hall. The study therefore shows that settings with higher ventilation rates and lower occupancy have lower transmission risk, but isolating the effect of occupancy is impossible in this case. The second of these studies, Telford *et al.* [24], used questionnaires to study long-term care facilities (LTCFs) and found that only 10% of LTCFs with a high prevalence of COVID-19 enforced maximum occupancy limits in enclosed spaces, while 64% of LTCFs with lower prevalence of COVID-19 enforced such restrictions. However, the different LTCFs also employed PPE, disinfection, and symptom screening in differing ways or at different rates and hence it was not possible to establish the effect of occupancy alone. Szablewski *et al.* [25] considered a sleep-away youth camp and found that the median SARS-CoV-2 attack rate was 50% (IQR 35–59%) when the median cabin occupancy was 11 and that the attack rate increased to 67% (IQR 54–72%) when the median cabin occupancy increased to 24. However, the different occupancy rates were observed at two different times of camp when the total numbers of infected people present were unknown. Close contact mixing also occurred outside the sleeping cabins, thus limiting the strength of the association. The final study by Baumgarte *et al.* [16] showed that in an outbreak caused by an infectious teacher who taught in four different classrooms, the classroom which had the highest transmission also had slightly higher occupancy rates ($5.33\;m^{3}$ room volume per person compared with $5.63\;m^{3}$, $6.33\;m^{3}$ and $6.33\;m^{3}$, respectively, in the other three classrooms). However, similar to the study by Walshe *et al.* [12], the setting with higher transmission and higher occupancy also had lower ventilation rates, preventing definitive quantitation of the effect of occupancy alone.

*No association*: One study (Wang *et al.* [21]) reported no association between occupancy rates and transmission. They used questionnaires and interviews to consider secondary transmission in 124 families. Crowding of the household (measured as the residential area *per capita*) was one of a number of different factors considered and using statistical analysis they concluded that this factor did not have a significant impact on transmission.

*Unclear association*: Oginawati *et al.* [11] concluded that decreasing occupancy rates (measured as the residential area *per capita*) was associated with increased transmission in a residential setting. However, the authors attributed this result to the likelihood (based on trends in the area studied) that larger houses generally contained more family members (i.e. more people in the same house), and therefore a larger susceptible population for each index case. The significance for the association between lower occupancy rates with higher transmission in this study was therefore confounded by the measure of occupancy used.

### (c) Surface disinfection

Five studies were identified that considered the effectiveness of surface disinfection on transmission of SARS-CoV-2: (i) three showed a positive association, i.e. enhanced disinfection was associated with reduced transmission and (ii) two studies showed no association between disinfection and transmission. These studies relied on data collected through questionnaires, interviews or site visits.

*Positive association*: Atnafie *et al.* [22] and Kerai *et al.* [23] considered healthcare settings. Atnafie *et al.* [22] used data collected through questionnaires to show that hospital workers in an institution which did not decontaminate high touch surfaces had 2.5 (AOR=2.52, 95% CI=1.12–5.65) times the risk of getting infected compared with workers from institutes that decontaminated high touch surfaces. Similarly Kerai *et al.* [23] compared healthcare workers (HCWs) via questionnaire from a healthcare centre who tested positive to SARS-CoV-2 after exposure to a COVID-19 positive person, to HCWs from the same healthcare centre who tested negative (control cases), and concluded that the risk of infection among HCWs in a hospital increased by 41% if the decontamination practices of high touch surfaces were perceived to be inadequate by the HCWs. However, no sampling of environmental surfaces was undertaken, which weakens confidence in the conclusions. Wang *et al.* [21] considered secondary infection rates in 124 families (with at least 1 COVID-19 positive patient) and found that daily disinfection using chlorine or ethanol-based products was 77% effective (OR=0.23, 95% CI 0.07–0.84) in reducing secondary transmission.

Both of the studies on healthcare settings (Atnafie *et al.* [22], Kerai *et al.* [23]) were based on the perceptions of HCWs of their own implementation of surface disinfection, rather than an objective assessment of the implementation of decontamination policies. Furthermore, in the study by Kerai *et al.* [23], it was unclear whether the control cases were actually in contact with a COVID-19 positive patient during the study duration. These issues mean that the level of confidence which can be ascribed to their conclusions is low.

*No association*: Guedes *et al.* [20] studied haemodialysis units through questionnaires and Telford *et al.* [24] studied long-term care facilities through virtual and in-person site visits. In both cases, they found no difference in transmission among the settings that implemented enhanced disinfection measures and settings that did not. These studies would have benefited from some actual environmental measurements such as surface samples for genomic SARS-CoV-2 or air flow measurements to complement the findings from the questionnaires.

### (d) Barrier devices

Only one of the identified studies considered the impact of barrier devices on transmission. Gettings *et al.* [17] (also considered above in §3a and i) found no association between the use of screens and transmission of SARS-CoV-2. They studied 169 school classrooms through surveys and found that the incidence of SARS-CoV-2 in schools that implemented barriers in some/no classrooms was similar to those which implemented barriers in all classrooms.

### (e) ${\mathrm{CO}}_{2}$ monitoring and one-way systems

No evidence was found for the effectiveness (or ineffectiveness) of the EC NPIs of ${\mathrm{CO}}_{2}$ monitoring and one-way systems in reducing transmission of SARS-CoV-2.

## 4. Risk of bias assessment

The risk of bias assessment for each domain across all included studies is given in table 3. Seventeen of the 19 studies were observational and two were laboratory controlled animal studies. Only studies that made a comparative analysis, either by means of before and after assessment or comparing similar settings, were included. However, in most studies, there was a critical or serious risk of bias in at least one domain. Many studies were assessed to have a critical risk of bias due to an inability to control for other NPIs, other environmental factors, non-adjustment of host factors including demographics, socio-economic status or susceptibility status. In the majority of studies, there was also a high risk of selection bias, where the selection of a certain group or environment was not clear or specified. There was also a high risk of information bias and bias in participant-reported outcomes. As a result, the quality of evidence was downgraded using the GRADE methodology for most comparisons.

## 5. Discussion

For all but one of the NPIs considered there were studies that reported a positive association (i.e. that the NPI reduced transmission), no association (i.e. that the NPI was ineffective for reducing transmission) and unclear association. Most of the studies were based on retrospective analyses of real-world settings with many factors either left uncontrolled or not measured including; the viral load of the infectors, the number of infectors, the size of the susceptible population, infection risk of the host outside the investigated setting, and the influence of other NPIs etc. [29]. These factors can influence the risk of transmission; for example, the viral emission in aerosols emitted from infected subjects during different respiratory activities such as breathing, talking and singing have been reported to vary between 0 and $10^{7}$ RNA copies per hour and the total volume of aerosols emitted is dependent on respiratory activity [30–36].

Due to such confounding factors, many of the studies reported in §3 have a high risk of bias.

Additionally, in most real-world settings, a combination of NPIs was generally employed together to reduce transmission, thereby making it difficult to determine the effect of a single NPI from observations of these settings. Many of the studies reported in §3 used questionnaires, interviews or surveys to collect data, thereby introducing potential bias in the data. For the few studies that measured the impact of the different NPIs in settings where there were recorded transmission events, the exact conditions (environmental, occupancy etc.) that were present during the transmission event were not fully documented, thereby weakening the confidence in the conclusions. Two laboratory animal studies were identified in this review. However, transmission between segregated infected and naive hamsters in controlled environments is not directly comparable to socially mixing humans. Furthermore, the sample size of these animal studies (of infectors and infectees) was very small, and there was a lack of measurement of factors such as the amount of virus present in the environment. The combination of these factors reduces the confidence of findings from these studies in terms of how they may relate to human transmission of SARS-CoV-2.

### (a) Additional evidence from studies that did not meet the strict criteria for the review

Studies that were published outside of the date range for the review or did not directly provide evidence for the effectiveness of the NPIs, but instead just suggested an association between the NPI and transmission, were excluded from the results section of this review. However, some of these studies do provide insights into the NPIs and are therefore briefly discussed here. The choice of which ‘excluded’ studies to discuss is not exhaustive, but chosen to represent the breadth of the material discovered in the literature.

*Ventilation and air filtration*: There are studies that report on outbreaks where, in the absence of adequate ventilation, the authors attributed long-range airborne transmission as the dominant transmission route [37–50]. They suggested that increased ventilation could reduce transmission. However, these studies did not make clear comparative assessments, making it challenging to confidently determine the effectiveness of this NPI. Indeed, two modelling studies of superspreading events (Miller *et al.* [37] and Vernez *et al.* [48]) showed that improving ventilation would not have significantly reduced transmission rates unless the length of exposure was also reduced. There are a number of reports of SARS-CoV-2 outbreaks in spite of adequate ventilation rates or the installation of air filtration units to improve air quality [51–57]. From these studies, one can infer that the viral load of the index case was particularly high or that other routes of transmission were important. One study reported increased ventilation as the likely cause for the transmission of the virus between two people residing in adjacent buildings that are placed unusually close to each other [58]. The authors attribute the transmission to the increased ventilation in the room of the infector which created a flow of contaminated air to the room of the infectee.

Some studies found a correlation between the probability of getting infected and the location of people relative to the air handling units in the setting [43,59]. These studies suggest that exposure to air flows from air handling units can enhance transmission or function as a direct route of transmission. However, these types of investigations did not take into account several confounders including but not limited to the risk of infection in another setting, the impact of other measures and human behaviour. Another study conducted in the setting of a cruise ship found no association between the location of cabins with respect to air handling units and the infection rates in cabins [60]. Finally, a study comparing an outbreak of influenza in a warship to an outbreak of COVID-19 in a cruise ship found that the warship with air filtration units and a relative negative pressure sick room contained the outbreak better than the cruise ship which had neither system. However, since the viruses are different and the combinations of other NPIs were different, definitive conclusions are not possible.

The analysis of air samples for SARS-CoV-2 genomic material (SARS-CoV-2 RNA by RT-PCR) is a method widely used for assessing the impact of ventilation and air cleaning devices. A number of studies have shown some reduction in SARS-CoV-2 genomic material in the air samples collected from hospitals with COVID-19 patients and enhanced ventilation [61,62]. HEPA filtration has also been shown to reduce the likelihood of detecting SARS-CoV-2 in air samples in bedrooms of SARS-CoV-2 positive isolating individuals [63]. However, in the study by Myers *et al.* [63], it was found that in some cases greater quantities of SARS-CoV-2 genomic material were detected in rooms with HEPA filters than those without. This may have been due to reasons such as different viral emission rates of the infectious occupants or the differences in the lengths of time individuals spent in the rooms. Parhizkar *et al.* [64] showed that enhanced ventilation and filtration significantly reduced aerosol and surface genomic viral loads of SARS-CoV-2 subjects housed in a controlled environment. Laboratory studies have also been able to demonstrate a reduction of SARS-CoV-2 genomic material when air cleaning technologies are employed and also demonstrate the removal of viable SARS-CoV-2 virus using a combination of HEPA filtration and UV (although amounts not quantified) [65,66]. However, demonstration of the presence or reduction of SARS-CoV-2 RNA may not directly inform our understanding of transmission because RNA is not a measure of viable virion. Quantification of viable virus in these types of studies could provide much better information about the effectiveness of a given NPI.

Buonanno *et al.* [67] considered the impact of EC NPIs on the transmission of SARS-CoV-2 but this was not included as it was published after the time window for the systematic review search. In this study, SARS-CoV-2 transmission in mechanically ventilated classrooms was significantly lower than in naturally ventilated classrooms. This difference was attributed to the hypothesized higher mechanical ventilation rate of $>1.4\;{l\;s}^{-1}\;\text{per person}$, with naturally ventilated classes all assumed to be ventilated lower than this threshold. However, they did not directly measure the ventilation rate in any of the classrooms and this assumption is not consistent with other studies that monitored naturally ventilated classrooms during the COVID-19 pandemic which observed acceptable ventilation rates [68,69]. Furthermore, several areas of confounders and bias were identified; for example, transmission outside of classrooms, localized geographical community infection rates in urban and rural areas were not accounted for, and a single infector was assumed to account for all other COVID-19 cases in the same classroom over a five-month period.

*Surface disinfection*: Contamination of surfaces in houses or hospital wards housing individuals with COVID-19 suggests that fomite transmission may be possible [70–72]. Lin *et al.* studied secondary transmission to HCWs that performed gastrointestinal endoscopy in 11 COVID-19 patients at a hospital in China, where enhanced disinfection strategies were in place both during and after the procedures. No SARS-CoV-2 transmission to HCWs was reported in the study [73]. However, multiple NPIs were employed in this setting (PPE, increased hand washing etc.) and it is therefore difficult to determine the effect of disinfection strategies alone. Nevertheless, it can be stated that the combination of NPIs identified in this study, which includes disinfection, appeared to reduce transmission to HCWs during endoscopies.

A number of studies involved the swabbing of surfaces in hospitals housing COVID-19 patients and were able to demonstrate reduction in the detection of SARS-CoV-2 genomic material after disinfection [74–79]. However, only one of these studies included the testing for viable virus and no evidence of viable SARS-CoV-2 was detected either before or after cleaning [75]. In some locations, there was evidence of increased genomic material after disinfection [79]. One drawback of using surface swabs is that this method may miss areas of contamination. In contrast to such in-room studies, *in vitro* disinfection of non-porous surfaces inoculated with inactivated SARS-CoV-2 demonstrated a reduction in detectable genomic material after disinfection [80].

*Barrier devices*: A type of barrier device (known by different names such as aerosol boxes, shields etc.) was used during aerosol generating procedures on COVID-19 positive patients in healthcare settings. There are studies that report zero or reduced transmission to HCWs during procedures where such barrier devices were employed [e.g. 81, 82]. However, the simultaneous presence of many other NPIs during such procedures (full PPE etc.) makes it difficult to determine the effectiveness of barrier devices on their own. In another study set in an office with poor ventilation, an outbreak from an index case was reported in spite of the presence of plexiglass screens between the workers [83]. This suggested that barrier devices did not provide adequate protection against transmission in an environment with poor ventilation and long occupancy periods. Interestingly, another study, again in an office setting, showed that certain kinds of barrier devices might in fact lead to increased transmission of SARS-CoV-2 [84]. They used a ${\mathrm{CO}}_{2}$ tracer decay method in an office which had a reported outbreak to show that the presence of screens in the office impeded ventilation, leading to stagnation of air in certain zones.

## 6. Conclusion

Evidence from the literature suggests that EC NPIs of ventilation, air cleaning devices and limiting room-occupancy may have a role in reducing transmission in specified settings. However, it is important to recognize that this conclusion is based on evidence which was usually of low or very low quality and hence the level of confidence ascribed to it is low. There were two significant challenges that limited the confidence in evidence for the effectiveness of many NPIs examined: (a) the low number of studies and (b) the low-quality assessment of the identified studies. What does this mean for the future? It is recommended that future studies on NPIs should be prioritized where there is a current lack of evidence on the effectiveness on transmission and where they have significant implementation cost including: (i) enhanced surface disinfection, (ii) use of barrier-devices, (iii) ${\mathrm{CO}}_{2}$ monitoring and (iv) one-way systems.

Many of the studies identified herein had a critical risk of bias mainly due to confounding factors. It is suggested that international level checklists/guidelines/protocols for both field and laboratory studies on pathogen transmission are established in order to ensure optimum utilization of available research resources. A more standardized approach focused on reducing confounding factors would equip future researchers with the tools that would enable a higher degree of confidence to be associated with their conclusions.

Only 19 studies from the initial dataset of 13 971 references addressed the issue of the effectiveness of EC NPIs on the transmission of SARS-CoV-2 in humans or animals (with two studies considering transmission in animals). The paucity and low quality of evidence makes it challenging to draw firm conclusions regarding the effectiveness of implementing these NPIs in the future to control the spread of SARS-CoV-2. This is exacerbated by apparently contradictory findings for almost all NPIs investigated (in that at least one study reported the opposite conclusion to the others). The review did not involve simply counting the number of studies for and against; the robustness of each study and findings were assessed and the extent to which confounding factors played a role was also considered. A majority of the studies were found to provide only low-quality evidence mainly due to the presence of many confounding factors in the study design.

The evidence identified for surface disinfection and barrier devices (screens) does not permit conclusions to be drawn regarding their effectiveness against SARS-CoV-2 transmission. While this does not mean that they are ineffective, their effect on SARS-CoV-2 transmission is not yet known. No studies were found that discussed the effect of ${\mathrm{CO}}_{2}$ monitoring or the implementation of one-way systems on the transmission of SARS-CoV-2. Further studies are required to judge the effectiveness of these NPIs.

Evidence, although of low quality, was found which showed increased ventilation and use of air cleaning devices reduced the transmission of SARS-CoV-2 in some situations. There was evidence, also of low quality, that decreasing the occupancy levels within some settings was found to be effective in reducing the transmission of SARS-CoV-2. An important caveat is that the evidence for these measures is limited to the settings that were studied and cannot necessarily be extrapolated beyond these. There is no evidence on the implementation of EC NPIs that provides any information on their effectiveness in altering transmission of SARS-CoV-2 at a community or population level.

In summary, this review has highlighted that there are significant knowledge gaps regarding the effectiveness of ECs in limiting transmission of SARS-CoV-2. It is extremely challenging to conduct controlled studies in the midst of a pandemic. However, it is important that lessons can be learned in these circumstances and protocols should be established to study the effectiveness of ECs using observational approaches during a pandemic. It is equally important that rigorous controlled studies are undertaken to study the effectiveness of ECs in experimental studies before another pandemic strikes.

## Data Availability

Additional information and the data are provided in electronic supplementary material [85].

## Appendix A

**Table 4.** Summary of the 19 papers which met the inclusion criteria for this review.

reference, year,		
country, setting, NPI	measurements and data	conclusions
Li *et al.* [10]	infection rate (percentage):	increased ventilation, but with lower adherence to masks, caused increased transmission
	**Dorm 1:** 74%	
**location:** China	**Dorm 2, Zone N:** 16–18%	
**setting**: residential	**Dorm 2, Zone M:** 8%	
**NPI of interest:**	ventilation:	
**EC:** ventilation	**Dorm 1:** $12.9\text{--}32.4\;m^{3}\;h^{-1}$	
**oth:** masks	**Dorm 2, Zone N:** $28\;m^{3}\;h^{-1}$	
	**Dorm 2, Zone M:** $7.7\;m^{3}\;h^{-1}$	
	masks (qualitative measurement):	
	**Dorm 1:** personnel wore no masks	
	**Dorm 2, Zone N:** personnel wore cloth masks, members wore no masks at night	
	**Dorm 2, Zone M:** personnel wore cloth masks, members wore masks	
	plot of ventilation rates VS infection rates: no clear trend observable.	
Oginawati *et al.* [11]	thirty-eight houses of COVID-19 survivors divided into three clusters based on transmission (measured as no. of COVID-19 positive family members VS total no. of family members):	(i) increased ventilation shows reduced transmission
**location:** Indonesia	low transmission: 0–50%	(ii) Increased personal space shows increased transmission. The authors attribute this trend to presence of larger total household members, and therefore larger susceptible population, in larger houses.
**setting:** residential	intermediate transmission: 50–90%	
**NPI of interest:** ventilation and occupancy	high transmission: 100%	
	ventilation and personal space (occupancy) measured using measuring tape; Pearson correlation coefficient calculated:	
	for ventilation r=−0.522	
	for personal space r=0.459	
Walshe *et al.* [12]	Outbreak in a mean processing plant with 111 confirmed positive asymptomatic cases (estimated attach rate of 38%) in five weeks. Four weeks after first case, 32 workers test positive, among which 16 (50%) were working in the boning hall. First three symptomatic workers were from the boning hall.	(i) increased transmission observed in the Boning hall with decreased ventilation and increased occupancy levels, in comparison to the abattoir
**location:** Ireland		
**setting:** meat-processing plant (MPP)		
**NPI of interest:**Ventilation and occupancy	air quality measurements (${\mathrm{CO}}_{2}$ concentrations, temperature, relative humidity, aerosol particle numbers) were compared between two regions:	
	**Boning hall** with a relatively high proportion: ${\mathrm{CO}}_{2}$ concentration and average aerosol particle concentration increased throughout the day (except during breaks). Average temperature was $100^{\circ }C$ and relative humidity 66%. Air re-circulation mechanism in place. ${\mathrm{CO}}_{2}$ decay suggests approximately 0.4--0.5 ACH	
	**Abattoir** with a relatively low proportion: ${\mathrm{CO}}_{2}$ concentration decreased throughout the day. Number of aerosol particles showed no significant change through the day. Average temperature was $180^{\circ }C$ and relative humidity 71%. Air extraction mechanisms that provides approximately 8 ACH (based on volume of air extracted) in place.	
	occupancy and screens differed between the two areas:	
	**boning hall:** occupancy $-5.1\;m^{3}$ floor area per person; screens in place	
	**abattoir:** occupancy $-15.2\;m^{3}$ floor area per person. Not clear if screens in place	
	performance of an air filter also measured based on the changes to air quality; however transmission difference before and after installation of the filter not considered	
	many other NPIs were also in place before the outbreak, but differences in transmission due to them not measured	
Atnafie *et al.* [22]	Four hundred and eighteen people were studied, with 78 (18.7%) people with reported confirmed COVID-19 exposure and 78 (18.7%) people with confirmed COVID-19 infection. Data on perceived exposure of the worker, training need, as well as adherence to different NPIs including PPE usage, hand-washing habits, decontamination of high touch surfaces, changing of masks were collected through questionnaires and statistically correlated to infection rates.	surface disinfection was found to reduce transmission in healthcare settings
**location:** Ethiopia		
**setting:** governmenthospitals and health centres		
**EC NPI**—surface disinfection		
	from statistical analysis they conclude that hospital workers in an institution that does not decontaminate high touch surfaces had 2.5 (AOR=2.52, 95% CI=1.12–5.65) times the risk of getting infected when compared to workers from institutes that decontaminate high touch surfaces	
Kerai *et al.* [23]	eighty-one HCWs with known COVID-19 exposure were considered as cases and 266 HCWs who were asymptomatic and tested negative for COVID-19 controls (no data on whether the control HCWs came in contact with COVID-19 positive patients); data collected by telephonic interviews	the practice of decontamination of high touch surfaces was found to be statistically correlated to reduced transmission
**location:** India		
**setting:** tertiarycare-dedicated COVID-19 hospital		
**NPI:** disinfection	the risk of infection was found to increase by a factor of 0.41 if high touch surfaces were not decontaminated	
Telford *et al.* [24]	twenty-four LTCFs totalling 2580 residents with 1004 (39%) residents infected with COVID-19 were included in the study	(i) maximum occupancy limits in enclosed places were observed in facilities with a lower prevelance of COVID-19 infections
**location:** USA	— higher-prevelance group (greater than 39% infection proportion) — 11 LTCFs	(ii) no significant differences in the adherance to disinfection strategies were observed in the groups with higher and lower prevalence of COVID-19 infections
	— lower-prevelance group (lower than 39% infection proportion) — 13 LTCFs	
**setting:** long-term care facilities (LTCFs)	adherence to NPIs of Hand Hygiene, Disinfection, Social Distancing, PPE and Symptom Screening were considered based on site visits (virtual or in-person)	
**NPI:** room occupancy, disinfection		
	maximum occupancy in small enclosed places (elevators, donning/doffing rooms etc.) was enforced in 10% of LTCFs in Higher prevalence group versus 64% in Lower-prevalence group, p=0.01	
	differences in adherence to disinfection between the higher and lower prevalence groups was not statistically significant	
Ou *et al.* [13]	an infected person travelled on two buses B1 and B2. Ventilation rates on the buses measured using tracer-decay and CFD	there was increased COVID-19 transmission on a bus with lower ventilation rates, compared to a bus with higher ventilation rates
**location:** China	—B1: **infected people** – 7/46 (and 1 more on a return trip without the index case)	
**setting:** buses		
**NPI: ventilation**	**ventilation rate** — 1.72 L/s per person.	
	**area occupied per passenger:** $0.6\;m^{2}$	
	— B2: **infected people** — 2/17.	
	**ventilation rate** — 3.22 $L\;s^{-1}$ per person	
	**area occupied per passenger:** $0.72\;m^{2}$	
Pokora *et al.* [14]	twenty-two meat and poultry plants with 19 072 employees studied (among which 880 were infected) 6552 employees from plants with many (greater than 10) infected were included for the statistical analysis. ventilation information was collected through questionnaires sent to the MPPs	the presence of a ventilation system was found to reduce the infection rates in meat processing plants
**location:** Germany		
**setting:** meat processing plants (MPPs)		
**NPI: ventilation**	they show that having a ventilation system reduced the chances of testing positive for COVID-19 (OR 0.388; 95% CI 0.299–0.503)	
Gettings *et al.* [17]	one hundred sixty-nine schools with 91 893 studies (556 infected) studied; data collected through surveys	(i) ventilation by air dilution reduced COVID-19 transmission
**location:** USA		
**setting:** classrooms	COVID-19 incidence (adjusted for Coutty level incidence) was impacted by different ventilation strategies. Incidence rates were:	(ii) ventilation by air dilution and purification/filteration reduced transmission more than when using dilution alone
**NPI:** ventilation and screens	— 39% lower in schools that reportedly improved ventilation	
	— 35% lower using air dilution (opening doors, opening windows or using fans) alone	
	— 48% lower using air dilution along with filtration/purification (HEPA filter and/or UVGI)	(iii) the use of screens does not impact transmission
	the COVID-19 incidence did not change significantly between schools that implemented barriers only in some/no classrooms compared to schools that implemented barriers in all classrooms	
Feathers *et al.* [18] (2022)	reported 10–20 in-patients during the time of the outbreak and 10–20 ward sta.. during any time	improved ventilation through opening windows and using extractor fans **may** have reduced the incidence of COVID-19
**location:** UK		
**setting:** hospices for palliative care patients	During the outbreak 26 patients and 30 sta.. were infected (measured by laboratory based RT-PCR testing). After the outbreak, implementation of following NPIs were advised:	
**NPI:** ventilation	— **EC NPI:** improved ventilation through opening windows and leaving extractor fans in treatment rooms on	
	— **other NPI:** universal staff masking and asymptomatic staff screening	
	After the measures implemented (adherence to the NPIs not measured), the number of COVID-19 cases decreased to zero in three weeks. However, the decrease was noticed when the national COVID-19 incidence was decreasing.	
Wang *et al.* [21]	Secondary transmission studied in 335 people from 124 families with at least 1 laboratory confirmed COVID-19 case. From 41 primary cases, the secondary attack rate was 23% (77/335). The effectiveness of many NPIs in reducing secondary transmission was studied. Data collected through questionnaires and interviews.	disinfection using chlorine or ethanol-based products was found to reduce secondary transmission in households
**location:** China		
**setting:** residential		
**NPI:** disinfection, ventilation, occupancy		
	for reducing secondary transmission:	
	— daily disinfection using chlorine or ethanol-based products was 77% effective in reducing secondary transmission (OR=0.23, 95% CI 0.07–0.84)	
	— crowding of the household was not found to be significant	
	— duration of ventilation was found to be significant in univariable analysis, but not in their multivariable logistic regression analysis	
Guedes *et al.* [20]	One hundred twenty-one Dialysis facilities studied. The facilities managed 20 984 patients among which 1093 were confirmed of having COVID-19. Four hundred fifty-nine HCWs tested positive as well. Data collected through questionnaires.	improvements in ventilation and disinfection **not** found to be associated with decreased detection of COVID-19 clusters in haemodyalysis units
**location:** Brazil		
**setting:** hemodialysis facilities		
**NPI:** ventilation, disinfection	presence of COVID-19 clusters (defined as occurrence of more than 1 COVID-19 case within 7 days during the same dialysis shift) was found not to be associated with a range of NPIs; this included cleaning and disinfection and ventilation	
Szablewski *et al.* [25]	six hundred twenty-seven people attended the camp and there were 351 COVID-19 positive cases (56% attack rate)	cabin occupancy rates may have been one of the factors that affected COVID-19 attack rates among campers
**location:** USA		
**setting:** sleep-away youth camp	— During orientation: occupancy—cabins with median occupancy of 11 (range 1–23 occupants). Attack rate—median cabin attack rate of 50% (interquartile range (IQR) =35--59%).	
**NPI:** occupancy	— During camp session: occupancy—cabins with median occupancy of 24 (range 1–26 occupants). Attack rate—median cabin attack rate of 67% (IQR=54--72%).	
Zhang *et al.* [28]	Tested a negative ionizer for reducing transmission in hamsters through direct contact and aerosol transmission. After housing three infected animals in the same cage with three naive animals for direct transmission study and with three naive animals separated by wire frames for aerosol transmission studies:	they found that the use of negative ionizer disinfection reduced aerosol transmission in hamsters
**location**, **setting:** Animal study	— without the ionizer 3/3 hamsters were infected	
**NPI:** air purification (negative ionizer)	— with the ionizer 3/3 hamsters were protected from aerosol transmission	
	— ionizer did not block direct contact transmission	
Nabirova *et al.* [15]	The study considered 296 PCR-positive cases and controls were 536 PCR-negative cases who lived in the same camp. Data collected through telephonic interviews. The statistical analysis showed that among environmental factors, only working in air-conditioned spaces showed a correlation with increased transmission, with other factors considered showing no significant association.	they found **no** significant correlation between COVID-19 transmission and working in ventilated workstations
**location:** Kazakhstan	— working in ventilated spaces was found to be not correlated with transmission (AOR 0.68 95% CI 0.36–1.24)	
**setting:** oilfield		
**NPI:** ventilation		
	occupancy was considered but not included in the analysis because of identified confounding factors	
Baumgarte *et al.* [16]	Three hundred sixty-eight students and 117 staff were tested, out of which 33 students (two students acquired infection from outside) and three staff tested positive. Data collected from the health department and school management and through telephonic interviews. Spatial conditions of classrooms obtained from building plans and local data. From one index case (staff A), the affected classroom from the most affected to the least affected were:	a school classroom with poorer ventilation and slightly higher occupancy rates saw higher transmission of COVID-19 from an index patient
**location:** Germany	— C.1 with 3h exposure time (ET)—16 cases, attack rate (AR) 57.14%	
**setting:** schools	— C.2 with 1:3hr ET—8 cases, AR 33.33%	
NPI: ventilation and room occupancy	— C.3 with 1:3hr ET—3 cases, AR 12.5% and	
	— C.4 with 0.45hr ET—1 case, AR 3.7%	
	all of the classrooms had lessons taught by the staff A with C.1 and C.2 having the class on day 3 and C.3 and C.4 on day 4 of the outbreak (staff A had improved personal protection measures on day 4)	
	in comparison to C.2, C.3 and C.4, the most affected class C.1 had	
	— poorer ventilation configuration (not measured but analysed through the structure of the available windows)	
	— and slightly higher occupancy ($5.3\;m^{3}$ room volume per person in C.1 compared to $6.3\;m^{3}$ is C.2 and C.3 and $5.6\;m^{3}$ in C.4)	
Cheng *et al.* [26]	compared two restaurant outbreaks R1 (in February 2021) and R2 (in December 2021)	(1) lower transmission observed in a restaurant with air purifiers installed and all diners vaccinated, when compared to a restraunt where no air purifiers were installed and all diners were unvaccinated
**location:** Hong Kong		(2) the restraunt with higher transmission also had lower occupancy rates
**setting:** restaurant	outbreak R2: 2.6% secondary attach rate from 1 index case:	
**NPI:** air purification	— ACH: 2.0	
	— air purifier: 14 UV-C air purifiers	
	— occupancy: $1.19\;m^{2}$ area per customer	
	— vaccination status: all present had two doses of COVID-19 vaccines	
	outbreak R1: 33.7% secondary attach rate (no. of index cases unknown):	
	— ACH: 1.2	
	— air purifier: none installed	
	— occupancy: $1.82\;m^{2}\;\text{per customer}$	
	— vaccination status: all present not vaccinated	
Fischer *et al.* [27]	Two donor hamsters inoculated with SARS-Cov-2 (two variants nCoV-WA1-2020 or hCoV-19/USA/KY-CDC-2-4242084/2021 (Delta)) were placed in a box separated by a tube from two naive hamsters. Two groups of set-ups were considered	the study found that UV-C light treatment reduced transmission in hamsters
**location/setting:** animal study	(1) with UV-C light treatment fitted inside the tube connecting the boxes that housed the donor and naive hamsters (representative of air treatment in a ducted system) and	
**NPI:** air purification	(2) no UV-C light treatment. Transmission was measured using qRT-PCR and enzyme-linked immunosorbent assay. After 4 h of exposure, all animals in the the no UV-C treatment group had detectable viable virus. And no virus was detected in the group with UV-C light treatment.	
Dancer *et al.* [19]	A window opening policy was enforced in three hospitals on 25th January 2021, along with multiple other NPIs that included daily surveillance, ward closures, universal masking, screening, restricting staff and patient movement and enhanced cleaning. Forty COVID-19 clusters were observed in three hospitals between 1st October and 25th January 2021, and only three clusters occurred between 25th January 2021, when window opening policy was implemented, and 31st March	window opening **may** have caused a decrease in outbreaks in hospital wards
**location:** Scotland		
**setting:** hospital wards		
**NPI:** ventilation	however, the decrease in outbreaks was observed when (1) the national COVID-19 incidence was decreasing, (2) when multiple NPIs were implemented together and (3) when the vaccination status of the population had changed	

## References

[1] UK Government. 2017 Pandemic flu guidance. https://www.gov.uk/guidance/pandemic-flu (accessed 31 January 2023).

[2] European Centre for Disease Prevention and Control. Eu influenza pandemic preparedness plans. https://www.ecdc.europa.eu/en/seasonal-influenza/preparedness/influenza-pandemic-preparedness-plans (accessed 31 January 2023).

[3] UKHSA. 2023 UK historical case numbers. https://coronavirus.data.gov.uk/details/cases (accessed 31 January 2023).

[4] UK Government. 2020 Prime minister address to the nation on coronavirus 23 march 2020. https://www.gov.uk/government/speeches/pm-address-to-the-nation-on-coronavirus-23-march-2020 (accessed 31 January 2023).

[5] BBC News. 2020 Coronavirus: latest patient was first to be infected in UK. https://www.bbc.co.uk/news/uk-51683428 (accessed 31 January 2023).

[6] Cevik M, Baral SD. 2021 Networks of SARS-CoV-2 transmission. Science (New York, NY) **373**, 162-163. (10.1126/science.abg0842) 34244400

[7] Cevik M, Baral SD. 2021 Networks of SARS-CoV-2 transmission. Science **373**, 162-163. (10.1126/science.abg0842) 34244400

[8] Scientific Advisory Group for Emergencies. 2021 Emg and nervtag: Update on transmission and environmental and behavioural mitigation strategies, including in the context of delta, 13 October 2021. https://www.gov.uk/government/publications/emg-and-nervtag-update-on-transmission-and-environmental-and-behavioural-mitigation-strategies-including-in-the-context-of-delta-13-october-2021 (accessed 31 January 2023).

[9] Wright L, Steptoe A, Fancourt D. 2020 Are we all in this together? Longitudinal assessment of cumulative adversities by socioeconomic position in the first 3 weeks of lockdown in the UK. J. Epidemiol. Commun. Health **74**, 683-688. (10.1136/jech-2020-214475) PMC7298206 32503892

[10] Li X, Yang F, Su Z, Liu L, Lin B. 2022 Aerosol transmission of SARS-CoV-2 in two dormitories - Hubei and Shandong Provinces, China, 2020. China CDC Wly **4**, 298-301. (10.46234/ccdcw2022.064) PMC9008262 35433092

[11] Oginawati K, Nathanael RJ, Pasaribu US, Mukhaiyar U, Humam A, Ilmi NFF, Susetyo SH. 2022 Analysis of the effect and role of indoor environmental quality in the COVID-19 transmission. Aerosol Air Qual. Res. **22**, 12. (10.4209/aaqr.210339)

[12] Walshe N *et al.* 2021 Assessment of environmental and occupational risk factors for the mitigation and containment of a COVID-19 outbreak in a meat processing plant. Front. Public Health **9**, 769238. (10.3389/fpubh.2021.769238) 34778195 PMC8578806

[13] Ou C *et al.* 2022 Insufficient ventilation led to a probable long-range airborne transmission of SARS-CoV-2 on two buses. Build. Environ. **207**, 108414. (10.1016/j.buildenv.2021.108414) 34629689 PMC8487323

[14] Pokora R *et al.* 2021 Investigation of superspreading COVID-19 outbreak events in meat and poultry processing plants in germany: A cross-sectional study. PLoS ONE **16**, e0242456. (10.1371/journal.pone.0242456) 34111143 PMC8191887

[15] Nabirova D *et al.* 2022 Factors associated with an outbreak of COVID-19 in oilfield workers, Kazakhstan, 2020. Int. J. Environ. Res. Public Health **19**, 16. (10.3390/ijerph19063291) PMC8955266 35328978

[16] Baumgarte S, Hartkopf F, Holzer M, von Kleist M, Neitz S, Kriegel M, Bollongino K. 2022 Investigation of a limited but explosive COVID-19 outbreak in a german secondary school. Viruses **14**, 04. (10.3390/v14010087) PMC8780098 35062291

[17] Gettings J *et al.* 2021 Mask use and ventilation improvements to reduce COVID-19 incidence in elementary schools – georgia, november 16-december 11, 2020. MMWR - Morb. Mort. Wkly Rep. **70**, 779-784. (10.15585/mmwr.mm7021e1) PMC8158891 34043610

[18] Feathers L, Hinde T, Bale T, Hyde J, Bird PW, Holmes CW, Tang JW. 2022 Outbreak of SARS-CoV-2 at a hospice: terminated after the implementation of enhanced aerosol infection control measures. Interface Focus **12**, 20210066. (10.1098/rsfs.2021.0066) 35261730 PMC8831080

[19] Dancer SJ, Cormack K, Loh M, Coulombe C, Thomas L, Pravinkumar SJ, Kasengele K, King M-F, Keaney J. 2022 Healthcare-acquired clusters of COVID-19 across multiple wards in a scottish health board. J. Hosp. Infect. **120**, 23-30. (10.1016/j.jhin.2021.11.019) 34863874 PMC8634690

[20] Guedes AR, Tavares BM, Assis DB, Freire MP, Madalosso G, Levin AS, Perdigao Neto LV, Oliveira MS. 2021 Statewide evaluation of infection control measures for preventing coronavirus disease 2019 in hemodialysis facilities. Clinics (Sao Paulo, Brazil) **76**, e3299. (10.6061/clinics/2021/e3299) 34644739 PMC8478131

[21] Wang Y *et al.* 2020 Reduction of secondary transmission of SARS-CoV-2 in households by face mask use, disinfection and social distancing: a cohort study in Beijing, China. BMJ Global Health **5**, 05. (10.1136/bmjgh-2020-002794) PMC7264640 32467353

[22] Atnafie SA, Anteneh DA, Yimenu DK, Kifle ZD. 2021 Assessment of exposure risks to COVID-19 among frontline health care workers in amhara region, ethiopia: a cross-sectional survey. PLoS ONE **16**, e0251000. (10.1371/journal.pone.0251000) 33914826 PMC8084207

[23] Kerai S, Singh R, Saxena KN, Desai SD. 2022 Assessment of risk factors for coronavirus disease-2019 in healthcare workers: a case-control study. Indian J. Crit. Care Med. **26**, 76-84. (10.5005/jp-journals-10071-24071) 35110849 PMC8783233

[24] Telford CT, Bystrom C, Fox T, Holland DP, Wiggins-Benn S, Mandani A, McCloud M, Shah S. 2021 COVID-19 infection prevention and control adherence in long-term care facilities, Atlanta, Georgia. J. Am. Geriatr. Soc. **69**, 581-586. (10.1111/jgs.17001) 33370463

[25] Szablewski CM *et al.* 2021 SARS-CoV-2 transmission dynamics in a sleep-away camp. Pediatrics **147**, 04. (10.1542/peds.2020-046524) PMC8982574 33504612

[26] Cheng VCC *et al.* 2022 Outbreak investigation of airborne transmission of omicron (b.1.1.529) SARS-CoV-2 variant of concern in a restaurant: implication for enhancement of indoor air dilution. J. Hazard. Mater. **430**, 10. (10.1016/j.jhazmat.2022.128504) PMC8848576 35739650

[27] Fischer RJ, Port JR, Holbrook MG, Yinda KC, Creusen M, Ter Stege J, de Samber M, Munster VJ. 2022 UV-C light completely blocks aerosol transmission of highly contagious SARS-CoV-2 variants WA1 and delta in hamsters. Environ. Sci. Technol. **56**, 12 424-12 430. (10.1021/acs.est.2c02822) PMC9437662 36001075

[28] Zhang C *et al.* 2022 Aerosol transmission of the pandemic SARS-CoV-2 and influenza a virus was blocked by negative ions. Front. Cell. Infect. Microbiol. **12**, 10. (10.3389/fcimb.2022.897416) PMC9105223 35573774

[29] Iddon C, Jones B, Sharpe P, Cevik M, Fitzgerald S. 2022 A population framework for predicting the proportion of people infected by the far-field airborne transmission of SARS-CoV-2 indoors. Build. Environ. **221**, 109309. (10.1016/j.buildenv.2022.109309) 35757305 PMC9212805

[30] Coleman KK *et al.* 2021 Viral load of severe acute respiratory syndrome coronavirus 2 (SARS-CoV-2) in respiratory aerosols emitted by patients with coronavirus disease 2019 (COVID-19) While breathing, talking, and singing. Clin. Infect. Dis. **2**, 1-7. (10.1093/cid/ciab691) PMC8436389 34358292

[31] Adenaiye OO *et al.* 2021 Infectious severe acute respiratory syndrome coronavirus 2 (SARS-CoV-2) in exhaled aerosols and efficacy of masks during early mild infection. Clin. Infect. Dis. **75**, e241-e248. (10.1093/cid/ciab797) PMC8522431 34519774

[32] Lai J *et al.* 2022 Exhaled breath aerosol shedding by highly transmissible versus prior SARS-CoV-2 variants. Clin. Infect. Dis. **76**, 786-794. (10.1093/cid/ciac846) PMC9620356 36285523

[33] Tan KS *et al.* 2023 SARS-CoV-2 omicron variant shedding during respiratory activities. Int. J. Infect. Dis. **131**, 19-25. (10.1016/j.ijid.2023.03.029) 36948451 PMC10028358

[34] Morawska L, Johnson GR, Ristovski ZD, Hargreaves M, Mengersen K, Corbett S, Chao CYH, Li Y, Katoshevski D. 2009 Size distribution and sites of origin of droplets expelled from the human respiratory tract during expiratory activities. J. Aerosol Sci **40**, 256-269. (10.1016/j.jaerosci.2008.11.002) PMC7126899 32287373

[35] Oswin HP *et al.* 2022 The dynamics of SARS-CoV-2 infectivity with changes in aerosol microenvironment. Proc. Natl Acad. Sci. USA **119**, e2200109119. (10.1073/pnas.2200109119) 35763573 PMC9271203

[36] Archer J *et al.* 2022 Comparing aerosol number and mass exhalation rates from children and adults during breathing, speaking and singing. Interface Focus **12**, 20210078. (10.1098/rsfs.2021.0078) 35261733 PMC8831083

[37] Miller SL *et al.* 2020 Transmission of SARS-CoV-2 by inhalation of respiratory aerosol in the Skagit Valley Chorale superspreading event. Indoor Air **31**, 314-323. (10.1111/ina.12751) 32979298 PMC7537089

[38] Katelaris AL, Wells J, Clark P, Norton S, Rockett R, Arnott A, Sintchenko V, Corbett S, Bag SK. 2021 Epidemiologic evidence for airborne transmission of SARS-CoV-2 during church singing, Australia, 2020. Emerg. Infect. Dis. **27**, 1677-1680. (10.3201/eid2706.210465) 33818372 PMC8153858

[39] Almilaji O. 2021 Air recirculation role in the spread of COVID-19 onboard the diamond princess cruise ship during a quarantine period. Aerosol Air Qual. Res. **21**, 11. (10.4209/aaqr.200495)

[40] Gu JLJ, Xu KLC, Lai WSZ, Zhou D, Xu CYB, Yang Z. 2020 Covid-19 outbreak associated with air conditioning in restaurant, Guangzhou, China, 2020. Emerg. Infect. Dis. **26**, 1628. (10.3201/eid2607.200764) 32240078 PMC7323555

[41] Li Y *et al.* 2021 Probable airborne transmission of SARS-CoV-2 in a poorly ventilated restaurant. Build. Environ. **196**, 107788. (10.1016/j.buildenv.2021.107788) 33746341 PMC7954773

[42] Kwon KS, Park JI, Park YJ, Jung DM, Ryu KW, Lee JH. 2020 Evidence of long-distance droplet transmission of SARS-CoV-2 by direct air flow in a restaurant in Korea. J. Korean Med. Sci. **35**, e415. (10.3346/jkms.2020.35.e415) 33258335 PMC7707926

[43] Wessendorf L *et al.* 2022 Dynamics, outcomes and prerequisites of the first SARS-CoV-2 superspreading event in Germany in February 2020: a cross-sectional epidemiological study. BMJ Open **12**, e059809. (10.1136/bmjopen-2021-059809) PMC8987213 35387836

[44] Wang Q *et al.* 2022 High attack rate in a Tong Lau house outbreak of COVID-19 with subdivided units in Hong Kong. Interface Focus **12**, 20210063. (10.1098/rsfs.2021.0063) 35261729 PMC8831081

[45] Cheng VCC *et al.* 2021 Nosocomial outbreak of coronavirus disease 2019 by possible airborne transmission leading to a superspreading event. Clin. Infect. Dis. **73**, E1356-E1364. (10.1093/cid/ciab313) 33851214 PMC8083289

[46] Rana K, Sharma B, Lakshmi PVM, Kaur M, Singh MP, Singh R, Aggarwal S, Biswal M. 2021 Nosocomial outbreak of SARS-CoV-2 in a non-COVID zone of a tertiary care hospital of north india: need to upgrade infection control practices. J. Primary Care Commun. Health **12**, 21501327211050753. (10.1177/21501327211050753) PMC8669871 34889120

[47] Jung JW *et al.* 2021 Nosocomial outbreak of COVID-19 in a hematologic ward. Infect. Chemother. **53**, 332-341. (10.3947/ic.2021.0046) 34216126 PMC8258301

[48] Vernez D, Schwarz S, Sauvain JJ, Petignat C, Suarez G. 2021 Probable aerosol transmission of SARS-CoV-2 in a poorly ventilated courtroom. Indoor Air **31**, 1776-1785. (10.1111/ina.12866) 34115411 PMC8597151

[49] Gunther T *et al.* 2020 SARS-CoV-2 outbreak investigation in a German meat processing plant. Embo Mol. Med. **12**, 10. (10.15252/emmm.202013296) PMC7646008 33012091

[50] Jones LD *et al.* 2022 Transmission of severe acute respiratory syndrome Coronavirus 2 (SARS-CoV-2) in a patient transport van. Clin. Infect. Dis. **74**, 339-342. (10.1093/cid/ciab347) 33893474 PMC8135457

[51] Fox-Lewis A, Williamson F, Harrower J, Ren XY, Sonder GJB, McNeill A, de Ligt J, Geoghegan JL. 2022 Airborne transmission of SARS-CoV-2 delta variant within tightly monitored isolation facility, New Zealand (Aotearoa). Emerg. Infect. Dis. **28**, 501-509. (10.3201/eid2803.212318) 34965365 PMC8888211

[52] Saidel-Odes L, Nesher L, Nativ R, Borer A. 2022 An outbreak of coronavirus disease 2019 (COVID-19) in hematology staff via airborne transmission. Infect. Control Hosp. Epidemiol. **43**, 405-407. (10.1017/ice.2020.1431) 33432896 PMC7853742

[53] Jones LD *et al.* 2022 Investigation of a cluster of severe acute respiratory syndrome coronavirus 2 (SARS-CoV-2) infections in a hospital administration building. Infect. Control Hosp. Epidemiol. **44**, 1-7. (10.1017/ice.2022.45) PMC9929709 35189996

[54] Sami S *et al.* 2022 Investigation of SARS-CoV-2 transmission associated with a large indoor convention – New York city, november-december 2021. MMWR - Morb. Mort. Wkly Rep. **71**, 243-248. (10.15585/mmwr.mm7107a4) PMC8853477 35176005

[55] de Man P, Paltansing S, Ong DSY, Vaessen N, van Nielen G, Koeleman JGM. 2021 Outbreak of coronavirus disease 2019 (COVID-19) in a nursing home associated with aerosol transmission as a result of inadequate ventilation. Clin. Infect. Dis. **73**, 170-171. (10.1093/cid/ciaa1270) 32857130 PMC7499506

[56] Chaintoutis SC *et al.* 2021 Outbreaks of SARS-CoV-2 in naturally infected mink farms: Impact, transmission dynamics, genetic patterns, and environmental contamination. PLoS Pathog. **17**, e1009883. (10.1371/journal.ppat.1009883) 34492088 PMC8448373

[57] Lam-Hine T, McCurdy SA, Santora L, Duncan L, Corbett-Detig R, Kapusinszky B, Willis M. 2021 Outbreak associated with SARS-CoV-2 b.1.617.2 (delta) variant in an elementary school – Marin County, California, May–June 2021. MMWR Morb. Mortal. Wkly. Rep. **70**, 1214-1219. (10.15585/mmwr.mm7035e2) 34473683 PMC8422870

[58] Zhang ZN *et al.* 2021 Field simulation of aerosol transmission of SARS-CoV-2 in a special building layout - Guangdong Province, China, 2021. China Cdc Wkly **3**, 711. (10.46234/ccdcw2021.176) 34594974 PMC8392788

[59] Jung J, Lee J, Park H, Lim YJ, Kim EO, Park MS, Kim SH. 2022 Nosocomial outbreak by delta variant from a fully vaccinated patient. J. Korean Med. Sci. **37**, 8. (10.3346/jkms.2022.37.e133) PMC9062276 35502502

[60] Xu P, Jia W, Qian H, Xiao S, Miao T, Yen H-L, Tan H, Kang M, Cowling BJ, Li Y. 2021 Lack of cross-transmission of SARS-CoV-2 between passenger’s cabins on the Diamond Princess cruise ship. Build. Environ. **198**, 107839. (10.1016/j.buildenv.2021.107839) 33875902 PMC8046742

[61] Thuresson S, Fraenkel CJ, Sasinovich S, Soldemyr J, Widell A, Medstrand P, Alsved M, Londahl J. 2022 Airborne severe acute respiratory syndrome coronavirus 2 (SARS-CoV-2) in hospitals: effects of aerosol-generating procedures, HEPA-filtration units, patient viral load, and physical distance. Clin. Infect. Dis. **75**, E89-E96. (10.1093/cid/ciac161) 35226740 PMC9383519

[62] Morris AC *et al.* 2022 The removal of airborne severe acute respiratory syndrome coronavirus 2 (SARS-CoV-2) and other microbial bioaerosols by air filtration on coronavirus disease 2019 (COVID-19) surge units. Clin. Infect. Dis. **75**, E97-E101. (10.1093/cid/ciab933) 34718446 PMC8689842

[63] Myers NT *et al.* 2022 Portable air cleaners and residential exposure to SARS-CoV-2 aerosols: a real-world study. Indoor Air **32**, 16. (10.1111/ina.13029) PMC9111720 35481935

[64] Parhizkar H, Dietz L, Olsen-Martinez A, Horve PF, Barnatan L, Northcutt D, Van den Wymelenberg KG. 2022 Quantifying environmental mitigation of aerosol viral load in a controlled chamber with participants diagnosed with coronavirus disease 2019. Clin. Infect. Dis. **75**, E174-E184. (10.1093/cid/ciac006) 34996097 PMC8755398

[65] Barnewall RE, Bischoff WE. 2021 Removal of SARS-CoV-2 bioaerosols using ultraviolet air filtration. Infect. Control Hosp. Epidemiol. **42**, 1014-1015. (10.1017/ice.2021.103) 33706834 PMC8007938

[66] Narayan R, Kundu D, Ghatak A, Tripathi S, Datta S. 2022 Efficient elimination of airborne pathogens: a study on aerosolized Mycobacterium tuberculosis and SARS-CoV-2 using ZeBox technology. J. Hosp. Infect. **129**, 17-21. (10.1016/j.jhin.2022.07.021) 35940288 PMC9354421

[67] Buonanno G, Ricolfi L, Morawska L, Stabile L. 2022 Increasing ventilation reduces sars-cov-2 airborne transmission in schools: a retrospective cohort study in Italy’s marche region. Front. Public Health **10**, 4922. (10.3389/fpubh.2022.1087087) PMC9787545 36568748

[68] Gilio AD, Palmisani J, Pulimeno M, Cerino F, Cacace M, Miani A, de Gennaro G. 2021 ${\mathrm{CO}}_{2}$ concentration monitoring inside educational buildings as a strategic tool to reduce the risk of SARS-CoV-2 airborne transmission. Environ. Res. **202**, 111560. (10.1016/j.envres.2021.111560) 34224708 PMC8253691

[69] Burridge HC *et al.* 2023 Variations in classroom ventilation during the covid-19 pandemic: Insights from monitoring 36 naturally ventilated classrooms in the UK during 2021. J. Build. Eng. **63**, 105459. (10.1016/j.jobe.2022.105459) PMC9664748 40477828

[70] Leal J *et al.* 2023 Patient and ward related risk factors in a multi-ward nosocomial outbreak of covid-19: Outbreak investigation and matched case–control study. Antimicrob. Resist. Infect. Control **12**, 21. (10.1186/s13756-023-01215-1) 36949510 PMC10031162

[71] Derqui N *et al.* 2023 Risk factors and vectors for SARS-CoV-2 household transmission: a prospective, longitudinal cohort study. Lancet Microbe **4**, e397-e408. (10.1016/S2666-5247(23)00069-1) 37031689 PMC10132910

[72] Cordery R *et al.* 2022 Transmission of SARS-CoV-2 by children to contacts in schools and households: a prospective cohort and environmental sampling study in london. Lancet Microbe **3**, e814-e823. (10.1016/S2666-5247(22)00124-0) 36029775 PMC9401977

[73] Lin X *et al.* 2020 Practical experience of endoscope reprocessing and working-platform disinfection in COVID-19 patients: a report from guangdong China during the pandemic. Gastroenterol. Res. Pract. **2020**, 9869742. (10.1155/2020/9869742) 33488698 PMC7790553

[74] Brune Z *et al.* 2021 Effectiveness of SARS-CoV-2 Decontamination and Containment in a COVID-19 ICU. Int. J. Environ. Res. Public Health **18**, 9. (10.3390/ijerph18052479) PMC7967612 33802332

[75] Choi HK, Cui C, Seok H, Bae JY, Jeon JH, Lee GE, Choi WS, Park MS, Park DW. 2022 Feasibility of ultraviolet light-emitting diode irradiation robot for terminal decontamination of coronavirus disease 2019 (COVID-19) patient rooms. Infect. Control Hosp. Epidemiol. **43**, 232-237. (10.1017/ice.2021.95) 33685546 PMC8160490

[76] Kim UJ, Lee SY, Lee JY, Lee A, Kim SE, Choi OJ, Lee JS, Kee SJ, Jang HC. 2020 Air and environmental contamination caused by COVID-19 patients: a multi-center study. J. Korean Med. Sci. **35**, e332. (10.3346/jkms.2020.35.e332) 32959546 PMC7505729

[77] Lesho E *et al.* 2022 Effectiveness of various cleaning strategies in acute and long-term care facilities during novel corona virus 2019 disease pandemic-related staff shortages. PLoS ONE **17**, e0261365. (10.1371/journal.pone.0261365) 35061676 PMC8782535

[78] Su WL, Lin CP, Huang HC, Wu YK, Yang MC, Chiu SK, Peng MY, Chan MC, Chao YC. 2022 Clinical application of 222 nm wavelength ultraviolet C irradiation on SARS CoV-2 contaminated environments. J. Microbiol. Immunol. Infect. **55**, 166-169. (10.1016/j.jmii.2021.12.005) 35094944 PMC8755561

[79] Zhang HL *et al.* 2022 SARS-CoV-2 RNA persists on surfaces following terminal disinfection of COVID-19 hospital isolation rooms. Am. J. Infect. Control **50**, 462-464. (10.1016/j.ajic.2022.01.014) 35108581 PMC8801058

[80] Krasnikova MS, Lazareva EA, Yatsentyuk SP. 2022 The effect of disinfectants on the SARS-CoV-2 RNA detection in swabs from various surfaces. Appl. Biochem. Microbiol. **58**, 932-937. (10.1134/S000368382208004X) 36540191 PMC9756920

[81] Erbas M, Dost B. 2021 Efficacy and safety of an aerosol box for percutaneous tracheostomy in patients with COVID-19 in an intensive care unit. Jcpsp, J. College Phys. Surg. - Pak. **31**, S79-S82. (10.29271/jcpsp.2021.supp1.s79) 34530535

[82] Kirti YK, Soni S, Yashveer JK, Anjali AR. 2022 Novel use of tracheostomy shield for emergency tracheostomy in COVID 19 Era. Indian J. Otolaryngol. Head Neck Surg. **74**, 3416-3419. (10.1007/s12070-021-02632-7) 34026594 PMC8128088

[83] Sarti D, Campanelli T, Rondina T, Gasperini B. 2021 COVID-19 in workplaces: secondary transmission. Ann. Work Exposures Health **65**, 1145-1151. (10.1093/annweh/wxab023) 34374745

[84] Yokogawa S, Ishigaki Y, Kitamura H, Saito A, Kawauchi Y, Hiraide T. 2022 Prevention of SARS-CoV-2 airborne transmission in a workplace based on CO_2_ sensor network. *medRxiv*, pp. 2022–03. (10.1101/2022.03.04.22271934)

[85] Madhusudanan A, Iddon C, Cevik M, Naismith JH, Fitzgerald S. 2023 Non-pharmaceutical interventions for COVID-19: a systematic review on environmental control measures. Figshare. (10.6084/m9.figshare.c.6707872) PMC10446906 37611631

